# Acquired resistance to immunotherapy in MMR-D pancreatic cancer

**DOI:** 10.1186/s40425-018-0448-1

**Published:** 2018-11-20

**Authors:** Zishuo Ian Hu, Matthew D. Hellmann, Jedd D. Wolchok, Monika Vyas, Jinru Shia, Zsofia K. Stadler, Luis A. Diaz, Eileen M. O’Reilly

**Affiliations:** 1 Department of Medicine, Memorial Sloan Kettering Cancer Center, New York, NY USA; 2 Division of Solid Tumor Oncology, New York, NY USA; 3 Parker Institute for Cancer Immunotherapy at Memorial Sloan Kettering, New York, NY USA; 4 Department of Medicine, Weill Cornell Medical College, New York, NY USA; 5 Department of Pathology, Memorial Sloan Kettering Cancer Center, New York, USA; 6 Department of Pathology, Weill Cornell Medical College, New York, NY USA; 7 David M. Rubenstein Center for Pancreatic Cancer Research, New York, NY USA

**Keywords:** Pancreatic cancer, Acquired resistance, Immunotherapy, Mismatch repair deficiency

## Abstract

**Background:**

MMR-D pancreatic cancer have been reported to respond to checkpoint inhibitor therapy. Here, we report the first case of acquired resistance to immunotherapy in MMR-D pancreatic cancer.

**Case presentation:**

A 45-year-old woman with unresectable MMR-D pancreatic cancer was initially treated with FOLFIRINOX, FOLFIRI, and stereotactic body radiation with stable disease burden. After 3 months, imaging showed progression of disease with an increase in CA19-9. She was subsequently enrolled in a clinical trial of an anti-PD-L1 antibody in combination with an IDO1 inhibitor. She demonstrated a partial response to therapy by RECIST 1.1 criteria with declining tumor markers. Twenty-two months after beginning immunotherapy, imaging revealed an increasing left ovarian cystic mass. There were no other sites of progressive disease. The patient underwent a total hysterectomy and bilateral salpingo-oophorectomy, appendectomy, omentectomy and pelvic lymphadenopathy. Pathology was consistent with a metastasis from the pancreas involving the endometrium and left ovary. Thereafter, the patient continued with PD-1 blockade therapy off protocol with no further progressive disease. Immune profiling showed high levels of CD8+ T cells and PD-1 positive immune cells infiltrating the tumor, with a moderate level of PD-L1 expression in both the immune cells and the tumor cells. Next generation sequencing found only the *KRAS* G12D and *RNF43* G659Vfs*41 mutations were retained from the pre-treatment tumor in the treatment-resistant tumor.

**Conclusions:**

This is the first report describing acquired resistance to immunotherapy in MMR-D pancreatic cancer with accompanying genomic and immune profiling. This case of oligoprogression in the setting of immunotherapy demonstrates the feasibility of localized treatment followed by continuation of immunotherapy to sustain ongoing response.

## Background

As checkpoint inhibitors have now entered broad use for the treatment of solid tumors, an increasing number of patients who initially respond to immunotherapy have been identified to develop acquired resistance. Such reports have been described in individuals with melanoma, non-small cell lung cancer (NSCLC), uterine leiomyosarcoma, and mismatch repair deficient (MMR-D) colorectal cancer (CRC) patients [1–7].

Pancreatic ductal adenocarcinoma (PDAC) has been largely refractory to single and combination checkpoint inhibitor therapy [8–10]. The tumor microenvironment of PDAC have been described to be largely immunosuppressive, with involvement of regulatory T cells, tumor-associated macrophages (TAMs), and myeloid-derived suppressive cells (MDSCs) [11–13]. Another contributing factor to PDAC’s immunotherapy resistance may be PDAC’s relatively low tumor mutation burden (TMB) and poor antigenicity, leading to impaired endogenous T cell response to the tumor [14]. TMB, in general, has been reported to have a significant correlation with objective response rate to PD-1 inhibition [15]. However, a rare subset of PDAC patients with MMR-D has been reported to have partial and complete responses to immunotherapy [1, 14]. MMR-D occurs at a frequency of < 1% of all PDAC patients and is typically associated with germline mutations in MMR genes, IHC loss of MMR expression, an elevated MSIsensor score, significantly prolonged survival times, and high TMB.

Here, we describe a patient with locally advanced MMR-D PDAC who had a partial response to checkpoint inhibitor therapy, but subsequently acquired resistance to therapy and developed a metastasis to the ovary. We evaluated tumor cell-intrinsic and extrinsic causes of acquired resistance in the metastatic tumor. We determined the tumor mutational profile before and after acquired resistance using next generation sequencing (NGS) and assessed PD-1, PD-L1, and CD8+ T cell levels in the immunotherapy-resistant tumor specimen.

## Case presentation

### Clinical course

**Fig. 1:**
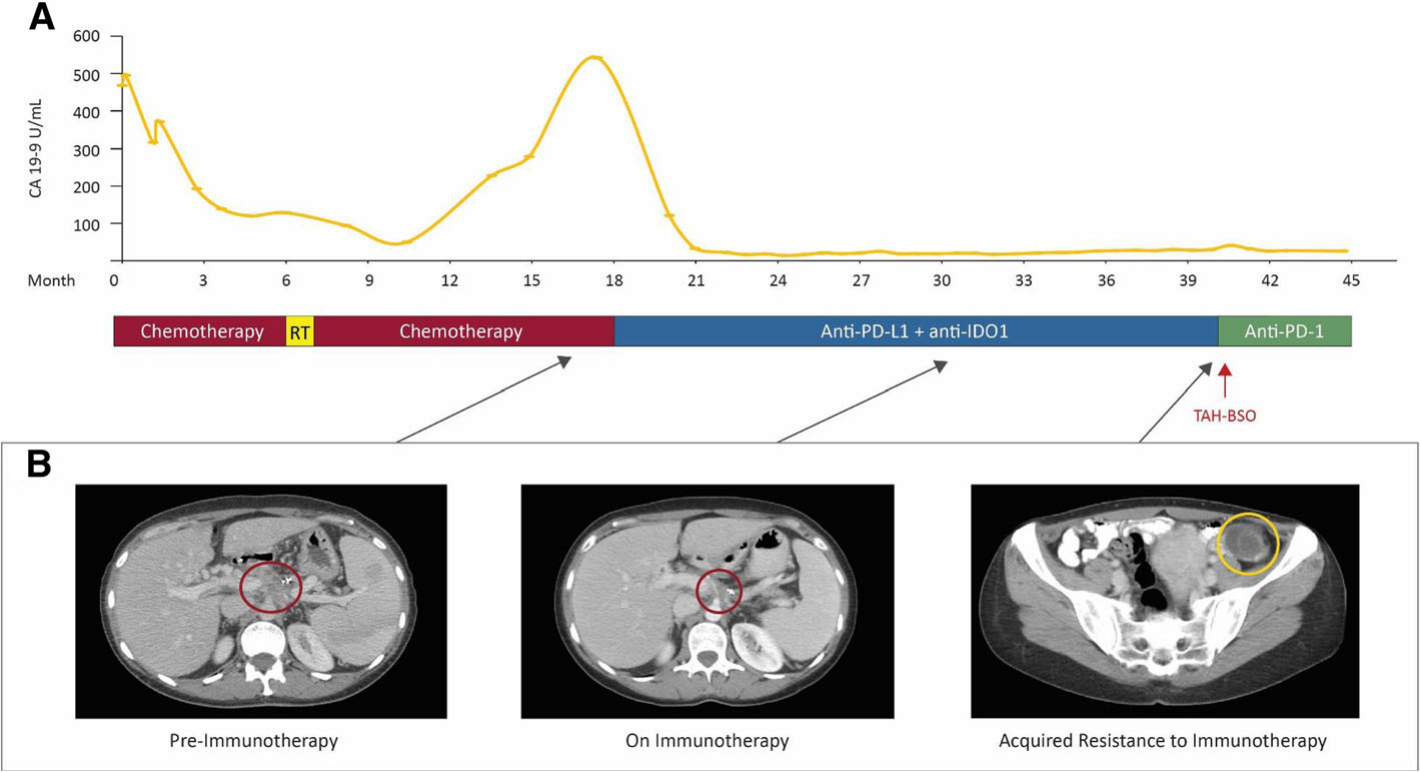
Clinical Pattern of Acquired Resistance. Panel **a** shows CA 19–9 levels corresponding to the timeline showing therapy. Panel **b** shows axial CT images corresponding to the primary pancreatic mass before treatment with immunotherapy and during immunotherapy, and the ovarian mass that developed after 22 months of immunotherapy. Red circles indicate the pancreatic mass and the yellow circle indicates the ovarian mass. Panel **b** shows the pancreatic mass after chemotherapy and RT

An otherwise healthy 45-year-old woman with known Lynch syndrome (germline mutation in *MLH1*) presented in 2014 with abdominal bloating. Computed tomography (CT) showed a 4 cm pancreatic body mass encasing the portal vein, splenomesenteric confluence, and common hepatic artery with enlarged periportal lymph nodes present. Biopsy revealed pancreatic adenocarcinoma. The patient was deemed to have unresectable disease and treated with FOLFIRINOX (5-fluorouracil, folinic acid, irinotecan, oxaliplatin) and FOLFIRI with stable disease burden and declining tumor markers (Fig. 1). She also received stereotactic body radiation therapy (SBRT) 3300 cGy in five fractions.

In 2015, CT scan revealed progression of disease, along with a rise in CA19-9 and clinical symptoms. The patient was enrolled in a clinical trial (NCT 02471846) of an anti-PD-L1 antibody in combination with an IDO1 inhibitor (navoximod). She demonstrated a partial response as defined by RECIST 1.1 criteria with declining tumor markers and prompt resolution of symptoms. In 2017, 22 months after beginning therapy, CT scan revealed an increasing left ovarian cystic mass. There were no other sites of progressive disease. The patient underwent a total hysterectomy and bilateral salpingo-oophorectomy, appendectomy, omentectomy and pelvic lymphadenopathy. Pathology was consistent with a metastasis from the pancreas involving the endometrium and left ovary. Thereafter, the patient continued with PD-1 blockade therapy off protocol with no further progressive disease.

### Genomic features of pre-treatment and treatment-resistant tumors

Tumor mutation profile and burden were determined through MSK-IMPACT, a next generation sequencing assay of somatic mutations in key cancer genes [16]. TMB was 50.2 mutations per megabase (mt/Mb) in the pretreatment sample and 21.1 mt/Mb in the acquired resistance sample (Table 1); both tumors were computationally consistent with microsatellite-instability high. Only the *KRAS* G12D and *RNF43* G659Vfs*41 mutations were retained from the pre-treatment tumor in the treatment-resistant tumor. No copy number alterations were detected in either the pre-treatment or the acquired resistance tumor sample. There was no loss-of-function mutations or loss of heterozygosity (LOH) in the *HLA* genes, *B2M, PTEN, JAK1, JAK2, or TAP1.*

**Table 1:** Mutations in primary and metastatic lesions

Gene	Protein change	Mutation type
Primary Pancreas Lesion		
*KRAS*	G12D	Missense_Mutation
*RNF43*	G659Vfs*41	Frame_Shift_Del
*ARID1A*	P1326Rfs*155	Frame_Shift_Del
*STK11*	P281Rfs*6	Frame_Shift_Del
*B2M*	S14Ffs*29	Frame_Shift_Del
*MAP3K1*	N1079Ifs*3	Frame_Shift_Del
*ARID1A*	A339Lfs*24	Frame_Shift_Del
*CIC*	P1529Lfs*91	Frame_Shift_Del
*ERCC4*	R689C	Missense_Mutation
*PIK3R1*	M1?	Translation_Start_Site
*KMT2A*	S2872*	Nonsense_Mutation
*BCL2L11*	S118Kfs*21	Frame_Shift_Ins
*MLH1*	X556_splice	Splice_Site
*FANCA*	P1444Rfs*3	Frame_Shift_Del
*BRCA2*	R2502C	Missense_Mutation
*BCOR*	S526 L	Missense_Mutation
*BRCA2*	K1191 M	Missense_Mutation
*ALK*	G1202Efs*56	Frame_Shift_Del
*ZFHX3*	A3407Lfs*78	Frame_Shift_Del
*PTPRS*	R1102H	Missense_Mutation
*FLT1*	R1305H	Missense_Mutation
*AR*	R280C	Missense_Mutation
*ROS1*	R1592C	Missense_Mutation
*JAK3*	P84Rfs*63	Frame_Shift_Del
*PTPRT*	I502T	Missense_Mutation
*RECQL4*	V155Sfs*25	Frame_Shift_Del
*VTCN1*	Y145H	Missense_Mutation
*RAF1*	Y458F	Missense_Mutation
*SETD2*	D1057N	Missense_Mutation
*GATA2*	P385S	Missense_Mutation
*APC*	R259Q	Missense_Mutation
*APC*	T1932A	Missense_Mutation
*ARID1B*	G314R	Missense_Mutation
*ARID1B*	A1002V	Missense_Mutation
*RECQL4*	G1166S	Missense_Mutation
*RECQL4*	A33V	Missense_Mutation
*PAX5*	R38C	Missense_Mutation
*TGFBR1*	V229D	Missense_Mutation
*H3F3C*	M120 V	Missense_Mutation
*KMT2D*	S1555F	Missense_Mutation
*CREBBP*	P2311L	Missense_Mutation
*ZFHX3*	T2667A	Missense_Mutation
*PLCG2*	L631R	Missense_Mutation
*SPOP*	N196K	Missense_Mutation
*STK11*	L285R	Missense_Mutation
*INSR*	R279H	Missense_Mutation
*MEF2B*	P106H	Missense_Mutation
*TOP1*	I457T	Missense_Mutation
*NF2*	K469R	Missense_Mutation
*AR*	V139M	Missense_Mutation
*TRAF7*	N174del	In_Frame_Del
Metastatic Site: Ovary/Endometrium		
*KRAS*	G12D	Missense_Mutation
*RNF43*	G659Vfs*41	Frame_Shift_Del
*TSC2*	Q35*	Nonsense_Mutation
*TP53*	R273C	Missense_Mutation
*STK11*	X245_splice	Splice_Site
*PBRM1*	P1411Lfs*21	Frame_Shift_Del
*BARD1*	K208Rfs*4	Frame_Shift_Del
*ATRX*	D1940Ifs*15	Frame_Shift_Del
*KMT2D*	K4318Efs*15	Frame_Shift_Del
*KMT2B*	G1879Vfs*16	Frame_Shift_Del
*NRAS*	A66V	Missense_Mutation
*CIC*	R440H	Missense_Mutation
*INSR*	R1331C	Missense_Mutation
*MYCN*	R285Q	Missense_Mutation
*BCL6*	S434 N	Missense_Mutation
*TNFAIP3*	K759Qfs*10	Frame_Shift_Ins
*RPS6KA4*	RPS6KA4-BAD fusion	Fusion
*FLT3*	S188R	Missense_Mutation
*ERBB2*	H193N	Missense_Mutation
*DOT1L*	V170 L	Missense_Mutation
*PTPRT*	E917V	Missense_Mutation
*HIST1H3F*	T119A	Missense_Mutation
*INHBA*	A41T	Missense_Mutation
*RXRA*	G73C	Missense_Mutation
*ARAF*	E556G	Missense_Mutation
*BAD*	RPS6KA4-BAD fusion	Fusion

### Pathological features of the treatment-resistant tumor

**Fig. 2:**
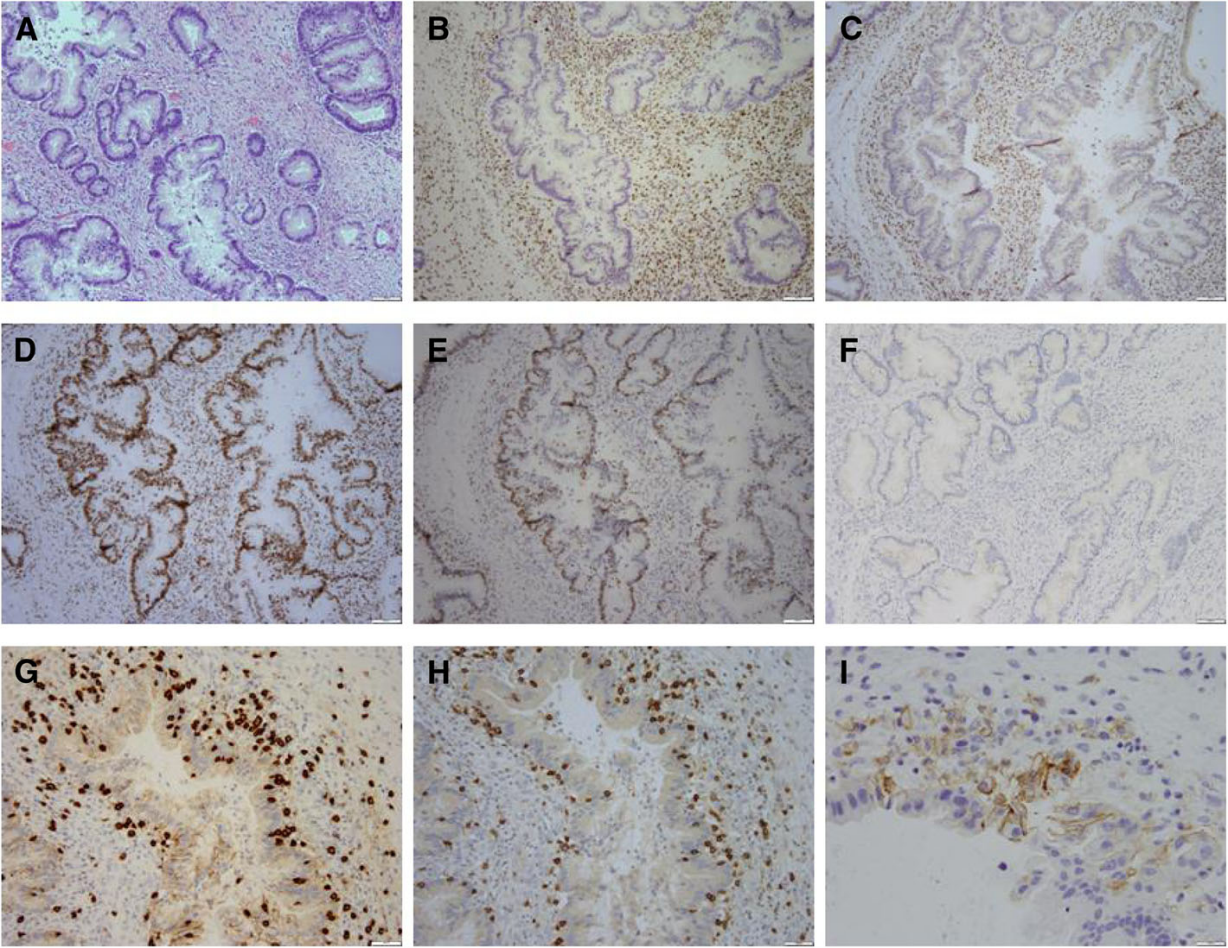
Immune Profiling of Metastatic Lesion. Metastatic pancreatic adenocarcinoma showing loss of MLH1 and PMS2 and increased immune cell infiltration. H&E section demonstrates a gland forming adenocarcinoma, morphologically compatible with pancreatic origin (**a**). By immunohistochemistry, the tumor cells show loss of staining for MLH1 (**b**) and PMS2 (**c**) and retained staining for MSH2 (**d**) and MSH6 (**e**). The tumor cells are also negative for PAX8 (**f**), in keeping with its non-Mullerian origin. Assessment of immune cell infiltration demonstrates florid CD8 positive T cells infiltrating the tumor epithelium and in the stroma surrounding the tumor epithelium (**g**). There is also prominent PD-1 positive immune cells (**h**) distributed similarly as the CD8 positive cells. PD-L1 expression is focally present in immune cells and in some tumor cells (**i**)

Immunohistochemistry (IHC) of the metastatic sample confirmed that the tumor was MMR-D, with loss of MLH1 and PMS2 expression (Fig. 2a-e). Histologically and immunophenotypically, the tumor exhibited features consistent with a metastasis of pancreatic origin including negative IHC staining for PAX8 (Fig. 2f), a marker typically associated with a Mullerian primary.

We were unable to assess immune cell infiltration with IHC in the pre-treatment tumor due to insufficient tissue. However, for the resected treatment-resistant metastasis, we found high levels of CD8+ T cells and PD-1 positive immune cells, with a moderate level of PD-L1 expression in both the immune cells and the tumor cells (Fig. 2g-i).

## Discussion

Patients treated with immunotherapy may respond durably, fail to respond, or initially respond but subsequently develop acquired resistance. Acquired resistance to immunotherapy is a consequence of a number of tumor-extrinsic and tumor cell-intrinsic factors [17]. Tumor-extrinsic acquired resistance can be due to insufficient CD8+ T cell infiltration at the tumor microenvironment (TME) and immunosuppression in the TME by regulatory T cells, MDSCs, and TAMs [18]. Mechanisms of tumor-intrinsic acquired resistance include decreases in and loss of neoantigens [2, 4, 19], disruption of neoantigen presentation [3, 5, 20, 21], and resistance to interferon gamma [5].

The ovaries have been previously reported as a potential sanctuary site for malignant gastrointestinal metastases given their resistance to chemotherapy [22]. In this case, however, we did not see a deficit in immune cell infiltration at the ovarian site. Given the abundant CD8+ T cell infiltration, PD-1, and PD-L1 expression in the ovarian site, we speculate that the resistance mechanism is driven less by tumor-extrinsic factors and more by tumor-intrinsic factors.

In this case of acquired resistance to PDAC, the decrease in tumor mutation burden after treatment is likely reflective of immunoediting [23–25]. However, the robust T cell infiltration within the resistant tumor microenvironment suggests a potential alternate mechanism restraining productive anti-tumor immunity. Through genomic profiling, we found no changes in loss of function or loss of heterozygosity in previously reported mechanisms of intrinsic resistance, including the *HLA* genes, *B2M*, *PTEN*, *JAK1*, *JAK2*, or *TAP1*. Similar cases in which the driver of resistance is unknown have been reported, and highlight the complexity of resistance in the context of immunotherapy and the need for larger, cooperative efforts to integrate analyses of these uncommon cases in order to reveal mechanistic insight [26].

In this PDAC patient, disease progression only occurred in the ovary, an uncommon site of metastases in PDAC [27]. The phenomenon and management of oligoprogression in the setting of acquired resistance to targeted therapy have been previously described in NSCLC [28]. But oligoprogression in the setting of acquired resistance to immunotherapy is less well described. A case series of acquired resistance to PD-1 axis inhibitors in 26 NSCLC patients found that a majority (89%) of these patients had recurrence limited to one or two sites of disease [7]. Isolated progression was also reported in the majority (78%) of 36 melanoma patients with acquired resistance to PD-1 blockade [29]. MMR-D patients under PD-1 blockade have been reported to develop acquired resistance, with tumors developing from occult sites such as the brain and the bone [1].

The present report has notable limitations. No clear mechanism of resistance was determined, although we speculate that immunoediting is a primary driving mechanism. Immunoediting is a dynamic dialogue between the immune system and the invading system that consists of elimination, equilibrium, and escape phases [30]. In the elimination phase, tumor cells are identified and eliminated by the immune system. In the equilibrium phase, the immune system is unable to eliminate all cancer cells but is able to contain further growth. In the escape phase, tumor cells variants are selected to proliferate in an immunologically intact environment. Genetic and epigenetic changes within these tumor cells grant additional resistance to immune elimination, allowing the tumor cells to grow. Further in vitro studies are needed to determine the specific acquired changes within the tumor and the selection pressure exerted by PD-L1 therapy. We also had insufficient pre-treatment tissue for immunopathologic testing to directly compare the phenotypic changes.

This is the first reported case, to our knowledge, of acquired immunotherapy resistance in PDAC with accompanying genomic and immune profiling of the metastasis. This case of oligoprogression in the setting of immunotherapy also highlights the feasibility of localized treatment followed by continuation of immunotherapy to sustain ongoing response elsewhere. A number of factors, including tumor heterogeneity, the specific resistance mechanism, and tissue-specific immunoregulation, likely influence the sites, extent, and rate of disease progression in acquired resistance to immunotherapy, and remain to be fully characterized [31].

## Abbreviations

CRC: Colorectal cancer
CT: Computed tomography
IHC: Immunohistochemistry
LOH: Loss of heterozygosity
MDSC: Myeloid-derived suppressive cell
MMR-D: Mismatch repair deficient
NGS: Next generation sequencing
NSCLC: Non-small cell lung cancer
PDAC: Pancreatic ductal adenocarcinoma
SBRT: Stereotactic body radiation
TAM: Tumor-associated macrophages
TMB: Tumor mutation burden
TME: Tumor microenvironment
T-regs: T-regulatory cells
